# Apical anatomy of mandibular second premolars at Nolla stage 8: A cross-sectional CBCT study in Chinese individuals

**DOI:** 10.1097/MD.0000000000050731

**Published:** 2026-09-11

**Authors:** Jingchao Han, Xiangyu Ma, Mingru Fan, Jianli Xie

**Affiliations:** a Department of Medical Imaging, Central Laboratory of Jinan Stomatological Hospital, Jinan Key Laboratory of Oral Tissue Regeneration, Jinan, Shandong Province, P.R. China; b Department of Prosthodontics, Central Laboratory of Jinan Stomatological Hospital, Jinan Key Laboratory of Oral Tissue Regeneration, Jinan, Shandong Province, P.R. China.

**Keywords:** apical foramen, cone-beam computed tomography, mandibular second premolars, measurement, Nolla stage 8, periapical radiolucency

## Abstract

This study used cone-beam computed tomography (CBCT) to measure the anatomical structures of the apical foramen of mandibular second premolars at Nolla stage 8. A total of 80 mandibular second premolars at Nolla stage 8 with deformed central cusp (DCC) deformity were selected as the observation group, and another 80 normal mandibular second premolars at the same stage were selected as the control group. The mesiodistal diameter (A-B) and buccolingual diameter (C-D) of the apical foramen, as well as the mesiodistal diameter (A1-B1), buccolingual diameter (C1-D1), and superoinferior diameter (E-F) of the periapical radiolucency, were measured using the built-in software of the CBCT system. The data were analyzed using SPSS 26.0 software. Results: In the observation group, the values of A-B, C-D, A1-B1, C1-D1, and E-F were (2.47 ± 0.26) mm, (3.28 ± 0.50) mm, (3.26 ± 0.30) mm, (4.25 ± 0.58) mm, and (3.06 ± 0.27) mm, respectively. In the control group, the corresponding values were (2.43 ± 0.29) mm, (3.27 ± 0.34) mm, (3.26 ± 0.41) mm, (4.24 ± 0.40) mm, and (3.28 ± 0.43) mm, respectively. Within each group, significant differences were found between A-B and C-D, as well as between A1-B1 and C1-D1 (*P* < .001). In both the observation and control groups, females exhibited significantly lower values than males for A-B, C-D, A1-B1, C1-D1, and E-F (*P* < .05). A-B and C-D were positively correlated with A1-B1, C1-D1, and E-F, respectively. This study confirmed that in Nolla stage 8 mandibular second premolars, the buccolingual diameters of both the apical foramen and periapical radiolucency were larger than the mesiodistal diameters, and females had smaller values for all parameters. Teeth with DCC but without apical periodontitis showed no significant anatomical differences from normal teeth. As the first CBCT-based comparison of apical anatomy between DCC and normal mandibular second premolars at this developmental stage, these results provide useful imaging references for early diagnosis and therapeutic monitoring of apical periodontitis caused by DCC fracture.

## 1. Introduction

**Figure 1.**
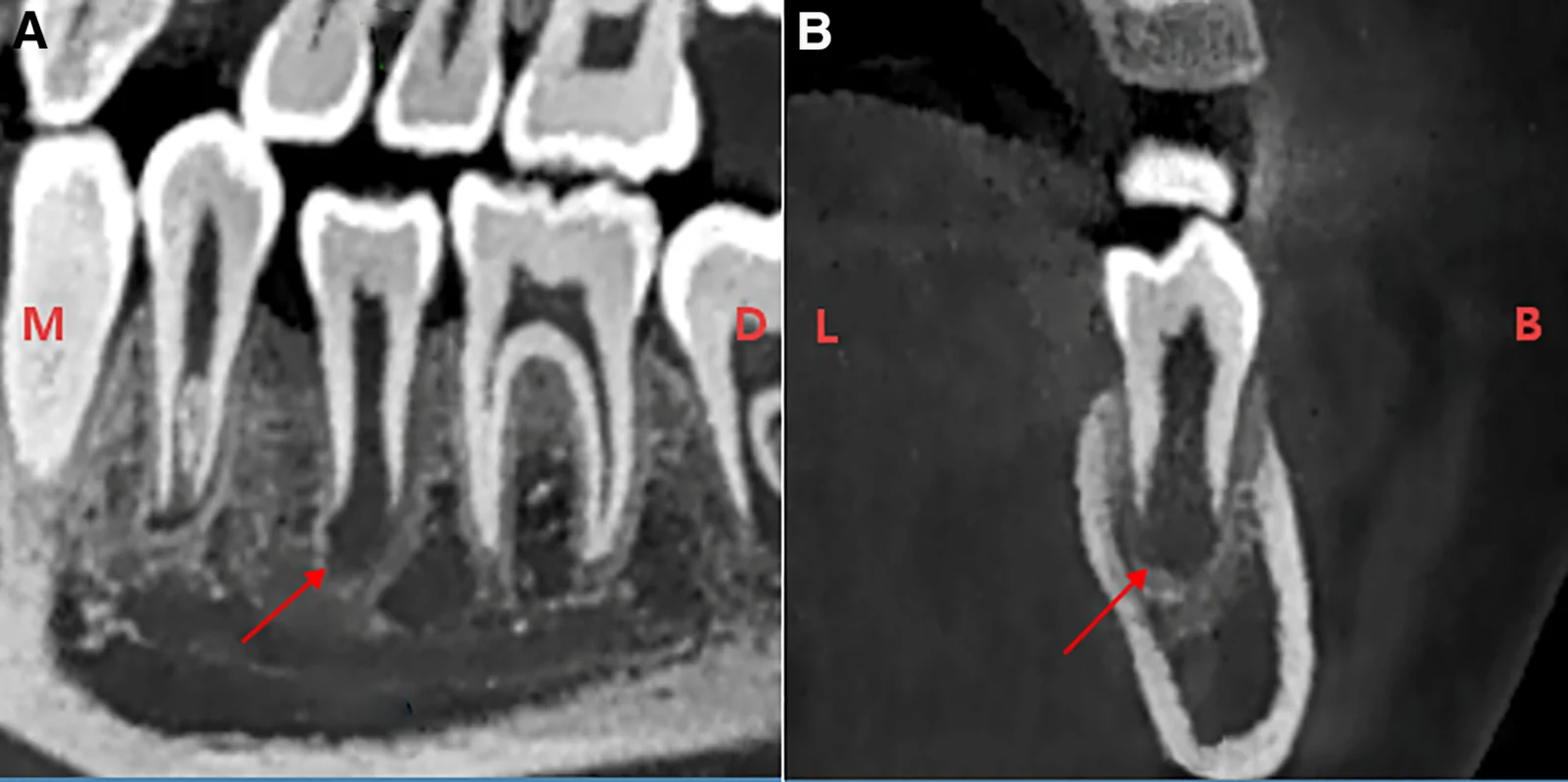
Mandibular second premolar at Nolla stage 8: (A) shows the mesiodistal section (M = mesial; D = distal), and (B) shows the buccolingual section (L = lingual; B = buccal). The root length reaches two-thirds of that of a mature root, the apical foramen is relatively wide and funnel-shaped, there is a radiolucent shadow in the apical region, and the surrounding high-density lamina dura is indistinct (red arrow).

In 1960, Nolla first divided the developmental process of each permanent tooth into 10 stages based on its radiographic appearance at different stages of development, with Nolla stage 8 defined as the stage when the root length reaches two-thirds of that of an adult tooth.^[1]^ The clinical eruption of mandibular second premolars typically occurs around 11 to 12 years of age, at which time most individuals are at Nolla stage 8. Radiographically, the apical foramen remains open, presenting a flared or funnel-shaped appearance. The residual dental papilla and dental follicle at the apical region appear as radiolucent areas on X-ray images, and the surrounding lamina dura is not yet fully developed (Fig. 1).^[2,3]^ Compared with Nolla stage 9 (near root maturation) and Nolla stage 10 (complete root maturation), the apical foramen at Nolla stage 8 is still open. Once external factors interfere and cause arrested root development, subsequent treatment becomes more challenging and the prognosis more uncertain.

**Figure 2.**
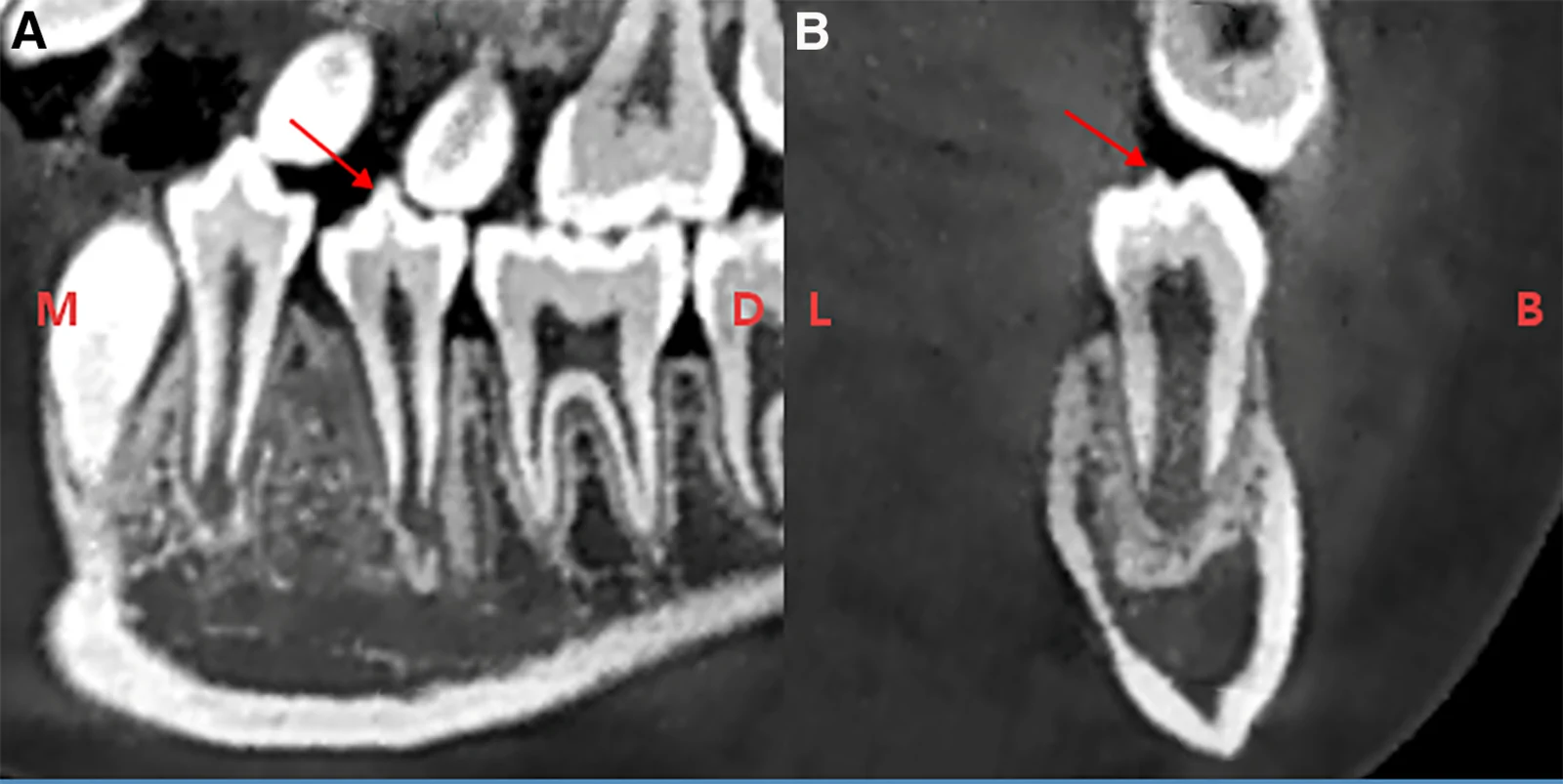
Mandibular second premolar with a central cusp deformity at Nolla stage 8: (A) shows the mesiodistal section (M = mesial; D = distal), and (B) shows the buccolingual section (L = lingual; B = buccal). The center of the pulp chamber roof projects toward the occlusal surface, and the central portion of the tooth’s occlusal surface shows an upward protruding cusp (red arrow).

The mandibular second premolar is the tooth with the highest incidence of deformed central cusp (DCC; Fig. 2). In some teeth, the anomalous cusp is accompanied by an extension of the pulp horn, making fracture of the dens evaginatus highly likely to lead to periapical periodontitis.^[4]^ Treatment for caries or periapical periodontitis in permanent teeth at Nolla stage 8 typically involves preventive and pulp-preserving approaches aimed at promoting continued root development. As periapical periodontitis progresses and the condition changes following treatment, the apical foramen and periapical radiolucency undergo certain changes.^[5,6]^ Furthermore, Nolla stage 8 represents a critical juncture in the development of mandibular second premolars, influencing the optimal timing for orthodontic intervention, the accuracy of eruption prediction, and the comprehensive assessment of associated pathological risks, thus serving as a key clinical “decision-making watershed.”^[7]^ Clinically, changes in the apical foramen and periapical radiolucency are used to diagnose early periapical periodontitis and to evaluate treatment outcomes.

Since its introduction into oral clinical practice in the 1990s, cone-beam computed tomography (CBCT) has been fully validated for obtaining precise measurements. Wang et al^[5]^ reported that it exhibits isotropic characteristics, enabling 1:1 imaging of anatomical structures. Despite the clinical significance of apical morphology in young permanent teeth, systematic data on mandibular second premolars at Nolla stage 8 remain scarce. Specifically, no previous study has provided sex-stratified normative reference values for this developmental stage, nor has the covariation between apical foramen dimensions and periapical radiolucency been quantified. To address these knowledge gaps, we employed CBCT with integrated NNT software to measure the apical foramen and periapical radiolucent shadow in Nolla stage 8 mandibular second premolars, with and without DCC. This study proposes the hypothesis that statistically significant differences exist in periapical anatomical features across groups, and that the corresponding quantitative reference values may enhance the diagnostic sensitivity for early detection and the accuracy of therapeutic planning.

## 2. Subjects and methods

### 2.1. Study subjects

This study was conducted after approval by the Ethics Committee of Jinan Stomatological Hospital (Approval No. JNSKQYY-2025-020). Given that this study was non-interventional and used de-identified imaging data, the Institutional Review Board waived the requirement for written informed consent. Sample size estimation was performed using G*Power 3.1.9.6 software based on an independent-samples *t*-test. With a statistical power of 80%, a 2-sided significance level α = 0.05, a pooled standard deviation of 0.3, and a target mean difference of 0.2, the calculated minimum sample size required for each group was 36 cases. To ensure adequate statistical power for stratified analyses, a total of 80 mandibular second premolars at Nolla stage 8 were finally included in each group for measurement and analysis.

From the imaging database of Jinan Stomatological Hospital, CBCT imaging data of subjects aged 10 to 12 years with mandibular second premolars at Nolla stage 8 were selected based on dental developmental characteristics, covering the period from January 2024 to February 2026. According to clinical records and imaging diagnostic reports, teeth with DCC were assigned to the observation group, while normally developing teeth served as the control group.

#### 2.1.1. Inclusion criteria

CBCT images of the mandibular second premolar met the criteria for Nolla stage 8. The mandibular second primary molar had exfoliated, and the second premolar had erupted normally. The diagnosis of central cusp deformity complied with standard criteria, with a prominent anomalous cusp visible on the occlusal surface, without fracture or attrition. The alveolar bone in the periapical region was well developed. Images were of sufficient clarity to meet measurement requirements.

#### 2.1.2. Exclusion criteria

Developmental abnormalities of the mandibular second premolar and/or the periapical alveolar bone. Concurrent infection or space-occupying lesions in the periapical region. Coexisting systemic diseases affecting tooth development, such as rickets or metabolic bone disorders.

### 2.2. Examination and measurement methods

Examinations were performed using a NEW TOM 5G-XL CBCT scanner (QR srl, Italy) with the following scanning parameters: 110 kV, 8.34 to 16.41 mA, Field of View: 10 cm × 10 cm to 19 cm × 16 cm, exposure time: 3.6–5.4 seconds, voxel size: 0.25 mm. Scanning protocol: patients were placed in the supine position with head-first entry, and images were acquired with the scanner rotating 360°. All images met the relevant quality control standards specified in American Association of Physicists in Medicine Task Group Report No. 261,^[8]^ with the following performance criteria: image uniformity within ±10.0%, spatial resolution ≥ 1.0 lp/mm, contrast resolution ≤ 2 mm, and geometric accuracy within ± 5.0%.

**Figure 3.**
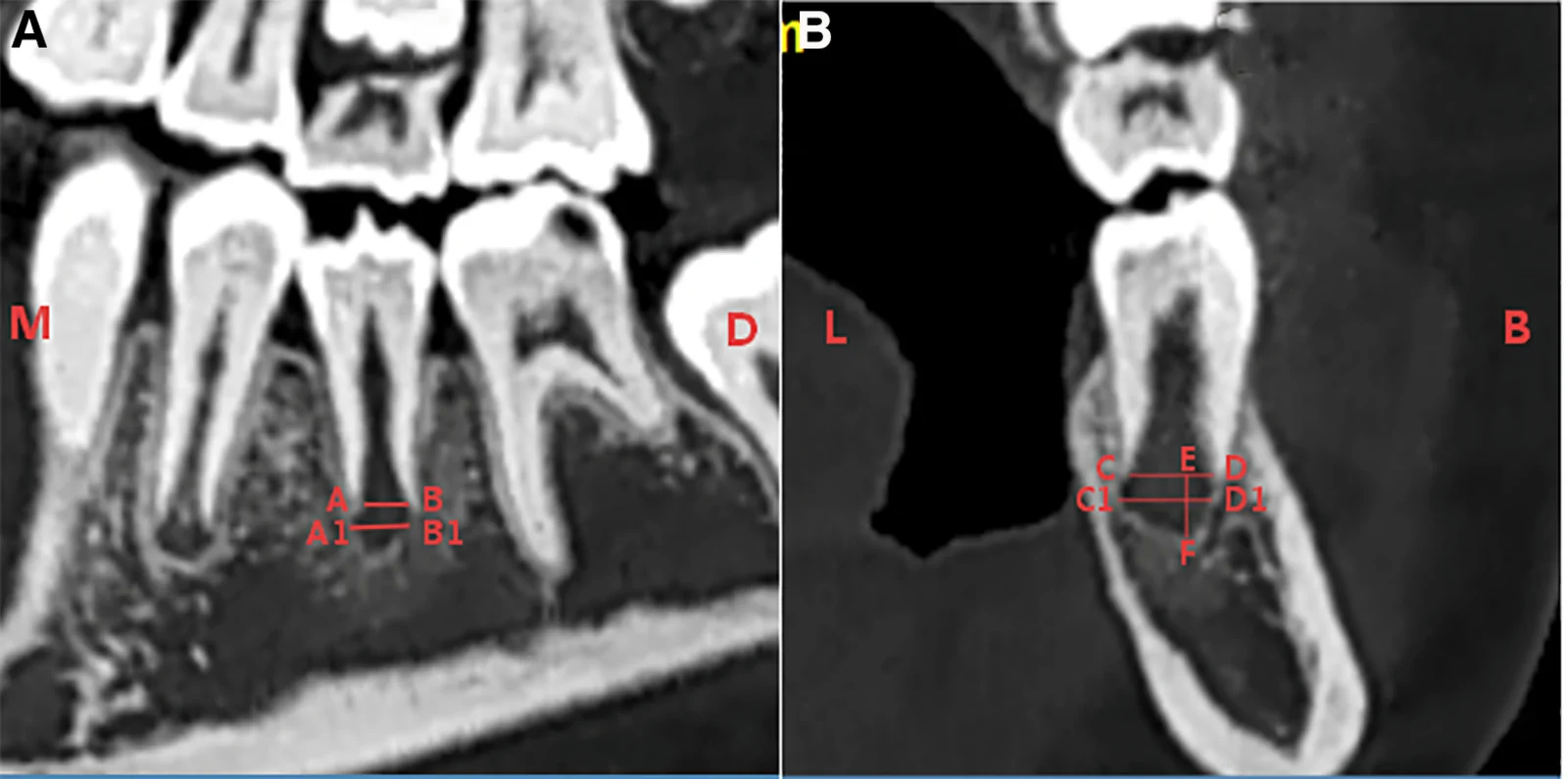
Schematic diagram of anatomical measurements of the mandibular second premolar apical region at Nolla stage 8. (A) Mesiodistal section: mesiodistal diameter of the apical foramen (A-B), mesiodistal diameter of the apical radiolucency (A1-B1). (B) Buccolingual section: buccolingual diameter of the apical foramen (C-D), buccolingual diameter of the apical radiolucency (C1-D1), and superoinferior diameter of the apical radiolucency (E-F).

All image measurements were performed using the proprietary NNT software integrated with the NEW TOM CBCT system. Multiplanar reconstruction (MPR) was applied for all assessments. Initially, the horizontal reference line was adjusted to be parallel to the occlusal plane, and the sagittal line was aligned with the long axis of the tooth. On the mesiodistal cross-sectional images, the section showing the largest periapical radiolucency was selected. The following parameters were measured for both the observation and control groups (Fig. 3A): A-B: mesiodistal diameter of the apical foramen – defined as the maximum mesiodistal distance between the mesial and distal inner walls of the apical foramen; A1-B1: mesiodistal diameter of the periapical radiolucency – defined as the maximum mesiodistal distance between the mesial and distal inner borders of the radiolucent shadow. On the buccolingual cross-sectional images, also at the level of the largest radiolucent area, the following measurements were recorded (Fig. 3B): C-D: buccolingual diameter of the apical foramen – defined as the maximum buccolingual distance between the lingual and buccal inner walls of the apical foramen; C1-D1: buccolingual diameter of the periapical radiolucency – defined as the maximum buccolingual distance between the lingual and buccal inner borders of the radiolucent shadow; E-F: superoinferior diameter of the periapical radiolucency – defined as the maximum vertical distance from the most superior inner border of the radiolucency to the horizontal level of the apical foramen.

All measurements were performed independently by 2 oral and maxillofacial radiologists, each with at least 5 years of clinical experience, who had received standardized training in the measurement protocol. In cases of complex images or disagreement between the 2 observers, a final decision was made by a senior oral and maxillofacial radiologist with over 15 years of experience.

### 2.3. Statistical methods

#### 2.3.1. Statistical analysis

Statistical analyses were performed using SPSS 26.0 (IBM Corporation, Armonk). Continuous data that passed normality tests were expressed as (*x̄* ± *s*), and categorical data as counts (percentages). Between-group comparisons of sex composition and age were conducted using the *χ*^2^ test and independent-samples *t*-test, respectively. To explore confounding by age, sex, and side on the 5 outcomes, multiple linear regression models were fitted for each outcome, with regression coefficients (B) and 95% confidence intervals (CI) quantifying effect sizes. Bonferroni correction was applied for 15 tests (α = 0.0033). Differences in each index between the observation and control groups were compared using independent-samples *t*-tests, while comparisons of different dimensions within the same root canal (A-B vs C-D; A1-B1 vs C1-D1) were performed using paired *t*-tests. Two-way ANOVA was used to test the interaction between group and sex (interaction *P* > .05 indicating no effect modification). Correlations among continuous indices were assessed using Pearson correlation coefficients. All tests were 2-sided, and *P* < .05 was considered statistically significant.

#### 2.3.2. Standard consistency assessment

Two researchers independently measured the relevant indices of 10 teeth, each obtaining 10 sets of data. One week later, both researchers repeated the measurements on the same 10 teeth. Bland–Altman analysis and intraclass correlation coefficient (ICC) were used to evaluate inter-observer (between the 2 researchers) and intra-observer (between the 2 repeated measurements by the same researcher) measurement consistency.

**Figure 4.**
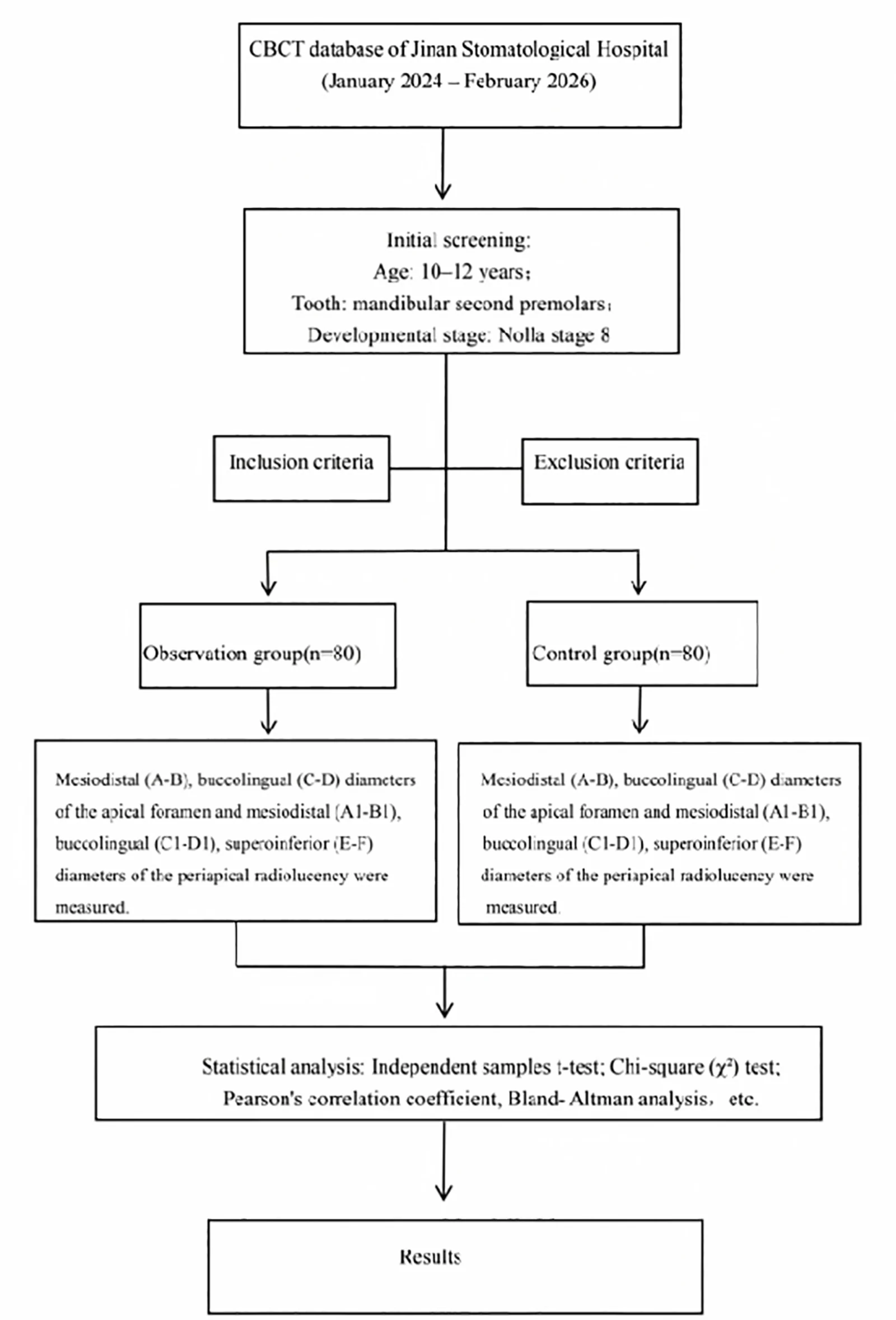
The diagrammatic representation of the chronology of the methodology. CBCT = cone-beam computed tomography.

The study flowchart is presented in Figure 4.

## 3. Results

### 3.1. Baseline characteristics of the 2 groups

The observation group included 37 males and 43 females, while the control group included 40 males and 40 females. There were no statistically significant differences in sex or age between the 2 groups (*P* > .05), indicating comparability, as shown in Table 1.

**Table 1:** Comparison of baseline characteristics between the 2 groups.

Group	n	Gender		Age (yr)
		Male	Female	
Observation	80	37	43	10.87 ± 0.42
Control	80	40	40	10.95 ± 0.50
*χ*^2^/*t*-value		0.225		0.964
*P*-value		.635		.337

### 3.2. Consistency assessment

**Figure 5.**
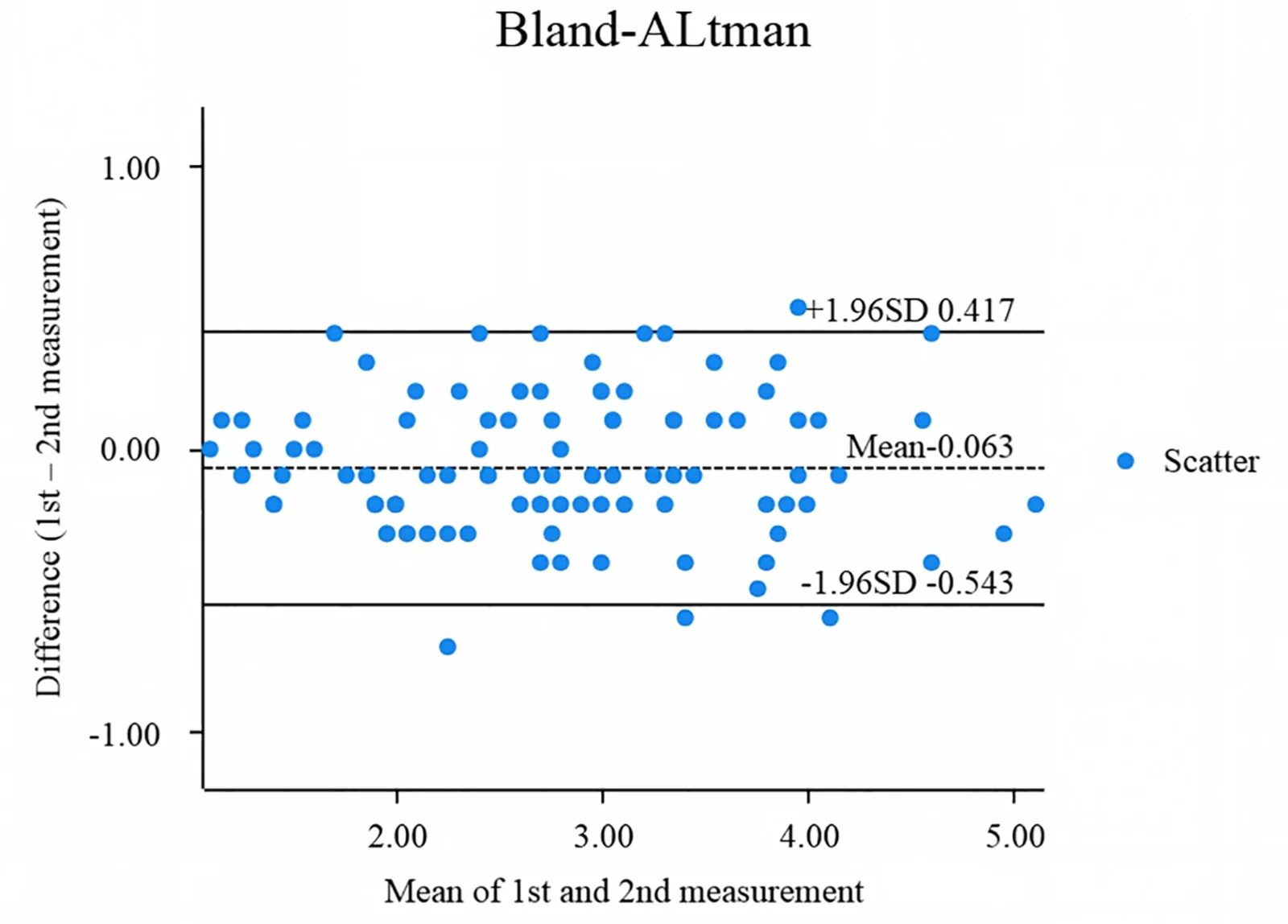
Bland–Altman plot for the first and second measurements.

**Figure 6.**
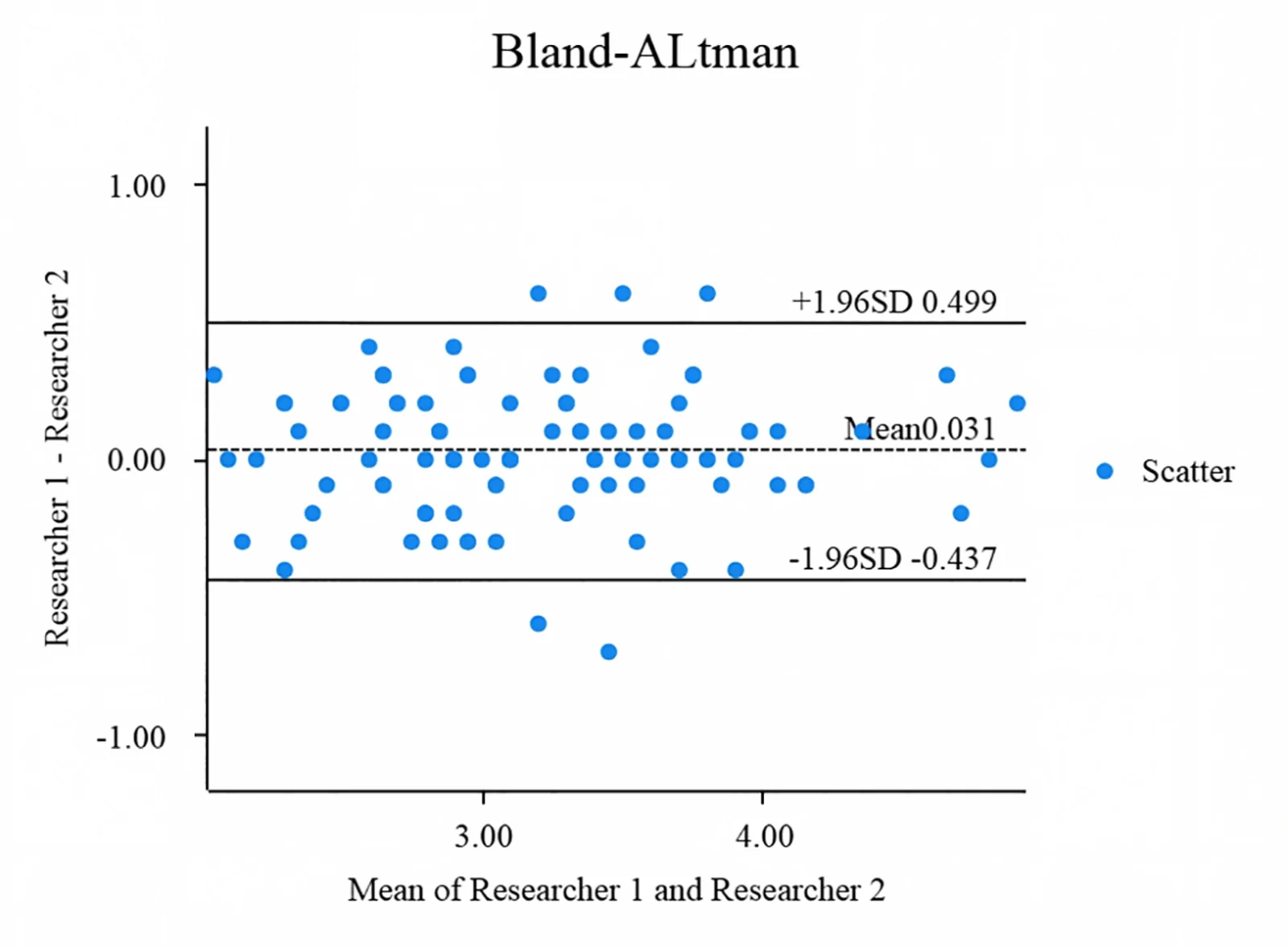
Bland–Altman plots for measurements by Researcher 1 and Researcher 2.

Intra-observer and inter-observer agreement were assessed using Bland–Altman analysis and the ICC. For intra-observer repeatability (Fig. 5) , the mean bias between 2 repeated measurements by the same examiner was −0.063 (95% limits of agreement (LoA): −0.543 to 0.417), and the ICC was 0.965, indicating excellent repeatability. For inter-observer reproducibility (Fig. 6) , the mean bias between the 2 examiners was 0.031 (95% LoA: −0.437 to 0.499), with 95.0% (95/100) of the differences falling within the LoA, and the ICC was 0.927, demonstrating substantial agreement. Both ICC values exceeded 0.90, confirming the high reliability and stability of the measurement protocol.

### 3.3. Measurement results

Multiple linear regression was performed to examine the potential confounding effects of age, gender, and side on 5 measurement indices. Gender was coded as a dummy variable (male = reference), side as right versus left, and age as a continuous variable. Bonferroni correction was applied for multiple comparisons (α = 0.0033). Gender showed a statistically significant association with A-B (*B* = −0.193, corrected *P* < .001), A1-B1 (*B* = −0.287, corrected *P* < .001), C1-D1 (*B* = −0.327, corrected *P* < .001), and E-F (*B* = −0.433, corrected *P* < .001), indicating that female participants had consistently lower values than males. For C-D, the gender effect was not significant after correction (raw *P* = .004, corrected *P* = .060). Age had no significant effect on any of the 5 outcomes (all corrected *P* ≈ 1.000), suggesting that within the current age range (10–12 years), age is not a notable confounder. Similarly, side (left vs right) did not significantly affect any measure (all corrected *P* > .05), indicating good bilateral symmetry. As shown in Table 2.

**Table 2:** Multiple linear regression analysis of age, gender, and side on 5 outcome variables.

Dependent variable	Independent variable	Coefficient (B)	Approx. 95% CI	Raw *P*-value	Bonferroni-corrected *P*-value*
A-B	Intercept	3.1248	(2.47, 3.78)	<.001	–
	Age	−0.0354	(−0.131, 0.060)	.474	1.000
	Gender (female vs male)	−0.1930	(−0.308, −0.078)	<.001	<.001
	Side (right vs left)	0.0030	(−0.081, 0.087)	.942	1.000
A1-B1	Intercept	3.7550	(2.91, 4.60)	<.001	–
	Age	0.0077	(−0.117, 0.133)	.903	1.000
	Gender (female vs male)	−0.2870	(−0.458, −0.116)	<.001	<.001
	Side (right vs left)	−0.0947	(−0.198, 0.008)	.072	1.000
C-D	Intercept	3.1575	(2.31, 4.00)	<.001	–
	Age	0.0231	(−0.110, 0.156)	.730	1.000
	Gender (female vs male)	−0.1798	(−0.303, −0.057)	.004	.060
	Side (right vs left)	−0.0542	(−0.164, 0.056)	.331	1.000
C1-D1	Intercept	4.7008	(3.58, 5.82)	<.001	–
	Age	−0.0834	(−0.232, 0.065)	.273	1.000
	Gender (female vs male)	−0.3269	(−0.521, −0.133)	<.001	<.001
	Side (right vs left)	−0.0106	(−0.133, 0.112)	.867	1.000
E-F	Intercept	5.6425	(4.04, 7.25)	<.001	–
	Age	−0.0653	(−0.234, 0.103)	.450	1.000
	Gender (female vs male)	−0.4331	(−0.690, −0.176)	<.001	<.001
	Side (right vs left)	−0.0187	(−0.160, 0.122)	.795	1.000

Corrected significance threshold α = 0.05/15 ≈ 0.0033. 95% CI = 95% confidence intervals.

CI = confidence interval.

* Bonferroni correction for 15 tests (5 outcomes × 3 predictors).

Independent-samples *t*-tests comparing the observation and control groups showed no statistically significant differences in any of the measured parameters (A-B, C-D, A1-B1, C1-D1, and E-F) between the 2 groups (all *P* > .05). Further paired *t*-tests were performed to compare different diameters within the same root canal (A-B vs C-D, and A1-B1 vs C1-D1). The results revealed that, in both the observation and control groups, the C-D values were significantly larger than the corresponding A-B values, and the C1-D1 values were significantly larger than the corresponding A1-B1 values (all *P* < .001). These findings indicate that, in Nolla stage 8 mandibular second premolars, the root canal and the periapical radiolucency both exhibit a buccolingual diameter that is greater than the mesiodistal diameter. As shown in Table 3.

**Table 3:** Comparison of measurement indices of apical foramen and periapical radiolucency between the 2 groups (*x̄* ± *s*) mm.

Parameter	Observation group (n = 80)	Control group (n = 80)	95% CI	*t*-value	*P*-value
A-B	2.47 ± 0.26*	2.43 ± 0.29‡	−0.054 to 0.117	0.721	.472
C-D	3.28 ± 0.50	3.27 ± 0.34	−0.120 to 0.154	0.186	.853
A1-B1	3.26 ± 0.30†	3.26 ± 0.41§	−0.111 to 0.116	0.044	.965
C1-D1	4.25 ± 0.58	4.24 ± 0.40	−0.138 to 0.173	0.223	.852
E-F	3.06 ± 0.27	3.05 ± 0.43	−0.098 to 0.128	0.262	.794

95% CI = 95% confidence intervals.

* Observation group – A-B vs C-D: *t* = 13.067, *P* = .000.

† Observation group – A1-B1 vs C1-D1: *t* = 13.545, *P* = .000.

‡ Control group – A-B vs C-D: *t* = 16.823, *P* = .000.

§ Control group – A-B vs C-D: A1-B1 vs C1-D1: *t* = 15.306, *P* = .000.

To systematically evaluate whether the sex effect was modified by group (observation vs control), a 2 (group) × 2 (sex) 2-way analysis of variance (ANOVA) was performed separately for the 5 measures. The results showed that the main effect of group was not statistically significant for any of the measures (all *P* > .05), which was consistent with the independent-samples *t*-test results and further confirmed the good comparability between the 2 groups. The main effect of sex was significant for A-B, A1-B1, C1-D1, and E-F (all *P* < .001), and also reached significance for C-D (*P* = .002), indicating that measurements were generally lower in females than in males. Importantly, the group-by-sex interaction was not significant for all 5 measures (all *P* > .05), indicating that the effect of sex on the measurements was consistent between the observation and control groups. As shown in Table 4.

**Table 4:** Two-way ANOVA of group and gender on 5 outcome variables.

Dependent variable	Source of variation	Sum of squares	df	Mean square	*F*-value	*P*-value
A-B	Group	0.035	1	0.035	0.521	.472
	Gender	0.975	1	0.975	14.552	<.001
	Group × Gender	0.054	1	0.054	0.806	.371
	Within (error)	10.456	156	0.067		
C-D	Group	0.005	1	0.005	0.031	.853
	Gender	1.578	1	1.578	9.801	.002
	Group × Gender	0.003	1	0.003	0.019	.891
	Within (error)	25.113	156	0.161		
A1-B1	Group	0.000	1	0.000	0.000	.965
	Gender	2.406	1	2.406	22.073	<.001
	Group × Gender	0.131	1	0.131	1.202	.275
	Within (error)	17.011	156	0.109		
C1-D1	Group	0.009	1	0.009	0.043	.852
	Gender	5.875	1	5.875	28.382	<.001
	Group × Gender	0.062	1	0.062	0.300	.584
	Within (error)	32.288	156	0.207		
E-F	Group	0.008	1	0.008	0.065	.794
	Gender	1.940	1	1.940	15.645	<.001
	Group × Gender	0.012	1	0.012	0.097	.752
	Within (error)	19.348	156	0.124		

Further subgroup analyses stratified by sex were performed for the observation and control groups separately. The results were consistent with those of the 2-way ANOVA: in both groups, females had significantly smaller values for A-B, C-D, A1-B1, C1-D1, and E-F than males (*P* < .05). As shown in Table 5.

**Table 5:** Comparison of measurement indicators of apical foramen and periapical radiolucency between gender subgroups (*x̄* ± *s*) mm.

Parameter	Observation group				Control group			
	Males (n = 37)	Females (n = 43)	95% (CI)	*P*-value	Males (n = 40)	Females (n = 40)	95% CI	*P*-value
A-B	2.53 ± 0.21	2.41 ± 0.28	0.013~0.238	.029	2.55 ± 0.29	2.31 ± 0.23	0.120~0.355	.000
C-D	3.44 ± 0.47	3.14 ± 0.48	0.094~0.519	.005	3.41 ± 0.31	3.12 ± 0.31	0.150~0.425	.000
A1-B1	3.36 ± 0.28	3.17 ± 0.30	0.055~0.315	.006	3.45 ± 0.38	3.06 ± 0.35	0.228~0.552	.000
C1-D1	4.46 ± 0.51	4.07 ± 0.58	0.139~0.631	.003	4.45 ± 0.34	4.02 ± 0.32	0.287~0.583	.000
E-F	3.14 ± 0.21	3.00 ± 0.31	0.018~0.257	.025	3.17 ± 0.36	2.92 ± 0.47	0.051~0.424	.013

95% CI = 95% confidence intervals.

Pearson correlation analysis revealed that A-B was significantly and strongly positively correlated with A1-B1 (*r* = 0.815), and C-D was similarly strongly correlated with C1-D1 (*r* = 0.897), both with *P* < .001, indicating a high degree of covariation between the apical foramen diameters and the corresponding periapical radiolucency diameters. In addition, both A-B and C-D showed moderate-to-weak positive correlations with E-F (*r* = 0.252–0.618, *P* ≤ .001), as shown in Table 6.

**Table 6:** Correlation analysis of measurement indices of apical foramen and periapical radiolucency.

Variable		A1-B1	C1-D1	E-F
A-B	*r* value	0.815	0.618	0.339
	*P*-value	.000	.000	.000
C-D	*r* value	0.538	0.897	0.252
	*P*-value	.000	.000	.001

## 4. Discussion

### 4.1. Biological basis for the lack of effect of DCC on apical development

The present study found no statistically significant differences between the observation and control groups in any of the measured diameters of the apical foramen and periapical radiolucency (*P* > .05), suggesting that the presence of DCC alone does not alter the normal developmental dimensions of the root apex at Nolla stage 8. The biological basis for this finding can be understood through the regulatory mechanisms of root development. Root development commences after crown formation, during which Hertwig’s epithelial root sheath (HERS) proliferates apically and induces dental papilla cells to differentiate into odontoblasts, thereby initiating dentin deposition.^[9,10]^ This process is precisely regulated by multiple signaling pathways, such as bone morphogenetic proteins (BMP), Wingless/Int-1 (Wnt), et al.^[11]^ Crown morphogenesis (including DCC), however, is primarily determined during the early patterning stage at the enamel-dentin junction. These 2 events are relatively independent in terms of their spatiotemporal expression. Peiris et al^[12]^ also pointed out that the relationship between crown size and root end complex size is not simply linear. The present study further corroborates this view from an imaging perspective, demonstrating that crown morphological variation, in the absence of secondary infection, does not reversely interfere with the programmed progression of root end development. However, Liu et al^[13]^ reported that the fracture rate of DCC reached 61.93% and increased with age. Once fracture leads to pulp exposure, inflammatory mediators can spread through the apical foramen to the root end complex, compromising the integrity of HERS and the proliferative and differentiation capacity of dental papilla cells, thereby resulting in arrested root development. Therefore, the threat posed by central cusp deformity to the root apex does not originate from the anomaly itself, but rather from the cascading infection triggered by its fracture.

### 4.2. Anatomical morphology of the apical region and clinical implications

The present study found that both the apical foramen and the periapical radiolucency exhibited an elliptical configuration, with significantly greater buccolingual diameters (C-D/C1-D1) than mesiodistal diameters (A-B/A1-B1), and the superoinferior diameter was the shortest. This morphological feature is consistent with the observations of Boreak et al^[14]^ and Pan et al^[15]^ on the horizontal morphology of mature mandibular second premolar root canals, and agrees with the findings of Moghadam and Sanei,^[16]^ who reported greater buccolingual than mesiodistal diameters at 3 mm from the root apex. These consistencies suggest that the apical morphology at Nolla stage 8 may predict the overall horizontal configuration of the root. From a developmental perspective, the elliptical shape of the root in horizontal cross-section, with a larger buccolingual diameter, can be attributed to differential rates of HERS proliferation and dental papilla cell differentiation between the buccolingual and mesiodistal directions. Specifically, odontoblastic activity and dentin deposition are more active in the buccolingual dimension, resulting in a wider buccolingual diameter.^[17]^

This finding suggests that during access cavity preparation, clinicians should avoid evaluating the root canal space solely from a mesiodistal perspective. Instead, preoperative planning should prioritize the buccolingual cross-sectional view on CBCT to adequately delineate the buccolingual extension of the canal, thereby reducing the risk of missed canals or incomplete instrumentation.

### 4.3. Developmental biological mechanisms of sex differences

Our multiple regression and subgroup analyses consistently showed that females had significantly smaller values for all measured diameters than males (*P* < .05), with no interaction between sex and grouping. The biological basis of this sexual dimorphism may involve multiple mechanisms. First, from a developmental timing perspective, tooth mineralization and eruption generally occur earlier in females than in males. Yang et al,^[18]^ in a survey of 3304 Chinese adolescents, demonstrated that the completion time of tooth development was significantly earlier in females than in males for all tooth positions except the central incisors and first molars. The mandibular second premolar, being one of the later-developing teeth in the permanent dentition, reaches Nolla stage 8 (10–12 years of age) precisely at the onset of the pubertal growth spurt. During this period, females generally lead males in both skeletal and dental development by approximately 1 to 2 years, resulting in a higher degree of mineralization and maturity in the apical region, which in turn manifests radiographically as smaller physiological dimensions of the apical foramen and periapical radiolucency. Second, with respect to the sexual dimorphism in tooth size, Alt et al^[19]^ reported that male teeth are generally larger than female teeth (male/female ratio: 1.01–1.08), and Leung et al^[20]^ also reported that in 12-year-old Chinese children, the mesiodistal diameter of the mandibular second premolar is greater in males than in females. The present study extends this dimensional dimorphism to the 3-dimensional anatomy of the apical region, demonstrating that sexual dimorphism is not only manifested in the crown but also exists in the apical region. Furthermore, the regulatory effects of sex hormones on the proliferative activity of dental follicle cells and dental papilla cells merit attention. Estrogen has been shown to accelerate the terminal differentiation and mineralization process of odontoblasts, which may result in a more “-convergent” – morphological appearance of the apical region in females at the same developmental stage.^[21,22]^

The core clinical implication of this finding is that sex-stratified normative reference values must be established for the apical region at Nolla stage 8. Failure to do so – misinterpreting the normally smaller values in females as developmental delay, or the normally larger values in males as pathological enlargement of the apical foramen – would directly compromise the accurate indication for apexification and the formulation of appropriate follow-up strategies.

### 4.4. Analysis of the covariation between apical foramen and periapical radiolucency

Pearson correlation analysis revealed that the diameters of the apical foramen were strongly and significantly positively correlated with their corresponding periapical radiolucency diameters (*r* = 0.815–0.897, *P* < .001), whereas the internal diameters of the apical foramen showed only weak correlations with the superoinferior diameter of the periapical radiolucency (*r* = 0.252–0.339). The histological basis for this covariation pattern lies in the fact that the periapical radiolucency at Nolla stage 8 appears on radiographs as a uniformly low-density radiolucent area, which anatomically corresponds to the incompletely mineralized developing root apical complex – comprising soft tissue components including the dental papilla, HERS, and dental follicle.^[23]^ As the apical foramen gradually narrows due to continuous dentin deposition (transitioning toward Nolla stages 9–10), the volume of the surrounding unmineralized tissue decreases accordingly, leading to a synchronous reduction in the radiographic radiolucent shadow. This coupling is most pronounced in the horizontal plane (mesiodistal and buccolingual dimensions) because centripetal dentin deposition is most directly reflected on the horizontal cross-section. In contrast, the superoinferior diameter represents the vertical height of the shadow, reflecting the extent of the unmineralized tissue along the long axis of the root. Its changes are driven not only by apical foramen narrowing but also by multiple factors such as periapical bone remodeling and HERS degeneration; thus, the covariation is relatively weak.^[10,23]^

This finding provides a quantitative tool for clinical differential diagnosis: during follow-up, if the enlargement of the horizontal diameters of the periapical shadow deviates significantly from the covariation trend with the apical foramen – for example, if the shadow enlarges markedly without appreciable changes in the apical foramen – this would be more suggestive of active apical periodontitis rather than a purely developmental manifestation.

### 4.5. Limitations of the present study

Several limitations of this study should be acknowledged. First, this was a retrospective study based on preexisting imaging data from a single center, and all participants were children aged 10 to 12 years from the Shandong region, with relatively homogeneous racial and geographic backgrounds. This may have introduced selection bias and may limit the generalizability of our findings to other regions or ethnic populations. Second, all measurements were performed using a single CBCT unit and its proprietary software; different devices, scanning parameters, or reconstruction algorithms may introduce systematic variations. We did not conduct cross-device or cross-software external validation; therefore, the normative reference values proposed in this study require further calibration through multicenter studies with larger sample sizes. Third, the retrospective design precluded us from obtaining information on certain potential confounding factors, such as detailed growth and development histories or nutritional status. Fourth, as a cross-sectional observational study, our findings demonstrate associations rather than causal relationships between the measured parameters. The observed covariation and sex-based differences should therefore be viewed as exploratory evidence that requires further validation, and our conclusions should be considered hypothesis-generating rather than confirmatory. Prospective longitudinal studies with larger and more diverse populations are needed to establish causal relationships and to validate the clinical applicability of our reference values across different settings. These limitations do not undermine the principal findings of this study; however, when translating the measurement results into clinical reference thresholds, careful consideration should be given to sample representativeness, measurement consistency, and the exploratory nature of the evidence.

## 5. Conclusions

In this study, CBCT was used to measure the apical anatomy of mandibular second premolars at Nolla stage 8, yielding reliable quantitative data. The results showed that, in the absence of periapical infection, the apical anatomy of teeth with DCC did not differ significantly from that of normally developing teeth, indicating that the presence of DCC alone does not interfere with apical root development at this stage. In all teeth, both the apical foramen and the periapical radiolucency exhibited a characteristic morphology, with buccolingual diameters consistently greater than mesiodistal diameters. Furthermore, females had significantly smaller values for all measured parameters than males. These findings underscore the necessity of sex-stratified normative assessment and reveal strong covariation between the apical foramen diameters and the corresponding periapical shadow diameters, thereby providing quantitative imaging references for the early differential diagnosis and therapeutic evaluation of periapical lesions during this developmental stage.

## Acknowledgments

The authors sincerely thank all medical staff who participated in the image measurement and data collection of this study.

## Author contributions

**Conceptualization:** Jingchao Han, Jianli Xie.

**Data curation:** Xiangyu Ma, Mingru Fan.

**Formal analysis:** Jingchao Han, Xiangyu Ma, Mingru Fan.

**Funding acquisition:** Jianli Xie.

**Investigation:** Jingchao Han.

**Methodology:** Xiangyu Ma, Mingru Fan.

**Project administration:** Mingru Fan.

**Resources:** Jianli Xie.

**Software:** Xiangyu Ma.

**Writing – original draft:** Jingchao Han, Jianli Xie.

**Writing – review & editing:** Jingchao Han, Jianli Xie.
